# Single‐Nucleotide Polymorphisms in Calcium and Vitamin D Maintenance Genes Association With Tooth Size

**DOI:** 10.1002/cre2.70472

**Published:** 2026-10-29

**Authors:** Lucas Cabral Rodrigues, Mirian Aiko Nakane Matsumoto, Maria Bernadete Sasso Stuani, Guido Artemio Marañón‐Vásquez, Fábio Lourenço Romano, Daniela Silva Barroso de Oliveira, Juliane Cora da Silva, Flares Baratto‐Filho, Christian Kirschneck, Erika Calvano Küchler, Caio Luiz Bitencourt Reis

**Affiliations:** ^1^ School of Dentistry Federal University of Alfenas Alfenas Minas Gerais Brazil; ^2^ Department of Pediatric Dentistry School of Dentistry of Ribeirão Preto, University of São Paulo Ribeirão Preto São Paulo Brazil; ^3^ School of Dentistry Tuiuti University of Paraná Curitiba Paraná Brazil; ^4^ Department of Orthodontics University Hospital Bonn, Medical Faculty Bonn Nordrhein‐Westfalen Germany

**Keywords:** calcium, genetic, orthodontic, pth, tooth size, vitamin D

## Abstract

**Objectives:**

This study investigated the association between Single Nucleotide Polymorphisms (SNPs) in calcium and vitamin D maintenance genes and mesiodistal tooth size (MTS) of permanent teeth.

**Materials and Methods:**

A total of 108 patients undergoing orthodontic treatment were recruited. Pretreatment orthodontic diagnostic casts were used to obtain the MTS. One single calibrated evaluator assessed the maximum crown dimensions in the mesiodistal direction of mandibular and maxillary permanent teeth with a digital caliper. Salivary genomic DNA was isolated from buccal cells, and six SNPs of the VDR, CYP24A1, CYP27B1, and PTH genes were selected. Real‐Time PCR was performed for the genotyping analysis. For statistics analysis, uni‐ and multivariate tests accounting for multiple observations per‐subject and adjusted by gender were performed (*α* = 5%).

**Results:**

In multivariate analysis, the rs694 and rs6256 (PTH) SNPs were associated (*p* < 0.05) with smaller MTS of the maxillary first molar. The rs7975232 (VDR) and rs307247 (PTH) were associated with a smaller mesiodistal size of the mandibular first molar. The rs7975232 (VDR) was also associated with a smaller mesiodistal size of the maxillary central incisor and a greater mesiodistal size of the maxillary first premolar. The rs4646536 (CYP27B1) was also associated with greater mesiodistal size of the first premolar.

**Conclusion:**

SNPs in the *PTH* and vitamin D‐related genes (*VDR* and *CYP27B1*) might be related to MTS variations of permanent teeth.

## 1 Introduction

Mesiodistal tooth size (MTS) has numerous practical implications in dentistry, particularly in orthodontics. Orthodontists consider MTS during orthodontic treatment planning due to its substantial impact on tooth alignment. The most common dental malocclusion is the discrepancy between MTS and arch length, which results in crowding or spacing (Lombardo et al. 2016). Predicting MTS of permanent teeth before their eruption allows orthodontists to anticipate potential challenges and promote less invasive and more comfortable treatments, such as arch expansion and space maintenance (Bitencourt Reis et al. 2023). Therefore, it is crucial to investigate the factors involved in MTS establishment and identify potential risk factors for crowding and spacing.

Genetic factors are consistently investigated as risk factors for crowding and spacing. Some specific genes, along with their promoters and enhancers, determine tooth size from the early stages of odontogenesis (Landin et al. 2012). Previous studies have already linked variations like Single Nucleotide Polymorphisms (SNPs) in odontogenesis‐related genes as modulators of permanent tooth size (Bitencourt Reis et al. 2023; Cunha et al. 2021a; Gerber et al. 2021). SNPs may affect the expression of proteins directly or indirectly involved in the differentiation and function of the odontogenic cells (odontoblasts and ameloblasts). Indirectly, SNPs in genes responsible for maintaining and providing essential organic substances for tooth development may impact the tooth size variation (Marañón‐Vásquez et al. 2021; Cunha et al. 2021b; Gerber et al. 2023).

Calcium and vitamin D are essential organic substances for tooth development (Reed et al. 2017; Mizumachi et al. 2017). A complex feedback system, comprising membranous receptors, enzymes, and hormones, regulates the body's levels of calcium and vitamin D. The vitamin D Receptor (VDR) is a nuclear receptor that binds to vitamin D and regulates its biological activity (Wang et al. 2012). CYP24A1 and CYP27B1 are enzymes involved in vitamin D metabolism. CYP24A1 is responsible for the degradation of active vitamin D, while CYP27B1 converts inactive vitamin D into its active form (Prosser and Jones 2004; Küchler et al. 2021; Herrmann et al. 2022). Additionally, the Parathyroid Hormone (PTH) is secreted by the parathyroid gland in response to low blood calcium levels to initiate bone absorption and rebalance the calcium levels in the body (Khundmiri et al. 2016).

Vitamin D‐related genes have been previously associated with various dentofacial phenotypes (Küchler et al. 2021; Herrmann et al. 2022). These genes might also influence calcium and vitamin D homeostasis during odontogenesis (Herrmann et al. 2022) and potentially impact MTS (Bitencourt Reis et al. 2023; Cunha et al. 2021a; Gerber et al. 2021; Marañón‐Vásquez et al. 2021; Cunha et al. 2021b; Gerber et al. 2023). While recent studies have reported associations between SNPs in genes directly involved with odontogenic cells, such as *SMAD6*, *RUNX2* (Bitencourt Reis et al. 2023; Gerber et al. 2021), *FGF10*, and *FG13* (Cunha et al. 2021a; Marañón‐Vásquez et al. 2021), the influence of genes involved in the maintenance of organic substances to tooth development, such as *VDR*, *CYP24A1*, and *CYP27B1* remains unexplored. To the best of our knowledge, only Gerber et al. (2023) investigated the impact of SNPs on the *PTH* gene. The identification of SNPs involved in MTS will improve the mechanism for predicting the MTS of permanent teeth before their eruption. Therefore, we aimed to evaluate the association between rs7975232 (*VDR*), rs927650 (*CYP24A1*), rs4646536 (*CYP27B1*), rs694, rs6256, and rs307247 (*PTH*) SNPs and MTS of permanent teeth.

## 2 Methods

Approval for this study was obtained from the Local Research Ethics Committee under protocol number 01451418.3.0000.5419/3.150.551. All patients included in this study and their legal guardians signed a written informed consent form following the Declaration of Helsinki.

### 2.1 Study Design

The STREGA (STrengthening the REporting of Genetic Association Studies) checklist was used to design and report this retrospective cross‐sectional study, as outlined in Little et al. (2009).

### 2.2 Setting and Participants

The study included patients undergoing orthodontic treatment at School of Dentistry of Ribeirao Preto at University of Sao Paulo in 2016 and 2018. Self‐reported Caucasian patients of both sexes, aged 7 years and older, who were not biologically related, without a history of previous orthodontic treatments and congenital syndromes or systemic diseases, were recruited. Patients with poor‐quality pretreatment dental casts and extreme tooth misalignment were excluded. This sample was previously described by Cunha et al. (2021b).

Sample size calculation was performed using PASS 2021 software (NCSS LLC, Kaysville, UT, USA). To estimate the effect size, parameters were obtained from Gerber et al. (2023), who studied the impact of SNPs in the *PTH* gene on tooth size in a similar demographic sample. We used the “Multiple Linear Regression” module in PASS, which indicates that a sample size of 96 achieves 80% power to detect an *R* ^2^ of 0.03 attributed to two independent variables (heterozygous and recessive genotypes) using an F‐Test with a significance level (alpha) of 0.05 adjusted for an additional covariate (biological sex).

### 2.3 Variables and Data Source Measurement

The MTS was characterized by the maximum distance between the mesial and distal anatomical proximal contact points of the tooth on a line perpendicular to the long axis of the tooth. Teeth with shape anomalies, Class I, II, or III restorations, interproximal caries or enamel reduction, severe occlusal dental wear, and fractured teeth were not evaluated. The MTS of each fully erupted permanent tooth was obtained from pretreatment dental casts using a digital caliper (Mitutoyo 500‐752‐20 Digimatic Digital Caliper) at a precision of 0.1 mm. Only one previously calibrated operator measured all maxillary and mandibular teeth, excluding the second and third molars.

The intraobserver reliability was previously evaluated during the MTS recording. Dental casts from 10 random subjects were selected, and the MTS of each fully erupted permanent tooth was obtained on two occasions, at least 2 weeks apart. The intra‐class correlation test was applied to the intraobserver reliability assessment, which ranged from 0.87 to 0.99 for each tooth.

Salivary samples were obtained for genomic DNA isolation from buccal cells following the protocol described by Küchler et al. (2012). The purity and concentration of the isolated DNA were quantified by spectrophotometer (Nanodrop 1000; Thermo Scientific, Wilmington, DE, USA). The genotyping was performed by real‐time polymerase chain reaction (PCR) using the Taqman method on the Prism QuantStudio 6 Flex PCR System (Applied Biosystems, Thermo Fisher Scientific Inc., Foster City, USA). The reaction contained 4 ng DNA/reaction, 1.5 μL Taqman PCR master mix, and 0.075 SNP assay (Applied Biosystems, Foster City, CA). SNPs in the *VDR*, *CYP24A1*, *CYP27B1*, and *PTH* genes were selected. In Table 1, there is a brief description of the selected SNPs and their association with previous studies that justify their use in this study.

**Table 1:** Details of studied SNPs.

**Gene**	**SNP**	**Base change**	**Previously reported effects**	**Genotyping Sucess rate**	***n* of dominant homozygous**	***n* of heterozygous**	***n* of recessive homozygous**	**HWE *p*‐value**
*VDR*	rs7975232	C > A	Vitamin D levels (Al‐Ghafari et al. 2019)	0.945	40	49	13	0.945
*CYP24A1*	rs927650	C > T	Vitamin D levels^38^	0.899	43	46	8	0.672
*CYP27B1*	rs4646536	A > G	Mandibular retrognathism (Küchler et al. 2021)	0.945	58	35	9	0.552
*PTH*	rs694	C > T	Tooth size (Gerber et al. 2023)	0.899	48	30	19	0.006
	rs6256	G > T	PTH levels (Tenne et al. 2008)	0.880	73	21	1	0.931
	rs307247	G > A	Tooth size (Gerber et al. 2023)	0.880	60	21	14	< 0.001

*Note:* Information's obtained in https://www.ncbi.nlm.nih.gov/snp and https://varsome.com/variant.

Abbreviation: SNP, Single Nucleotide Polymorphism.

### 2.4 Bias

Only one operator assessed the MTS to reduce variation. The operator was blinded to the patient's sex. Five dental casts per day were analyzed during the 4 months to prevent operator fatigue. Each tooth was measured three times, and the arithmetic means were calculated for subsequent analyses. If measurements differed by more than 0.2 mm, the measurements were repeated. The PCR analysis was performed blindly, and a 10% sample was genotyped twice to assess agreement, which was 100%. The statistical analysis, accounting for multiple observations per subject, was used to assess intra‐ and inter‐individual variability.

### 2.5 Statistical Methods

The Hardy–Weinberg equilibrium was calculated for each SNP through Pearson's chi‐square test (wpcalc.com).

The arithmetic mean of the MTS of each tooth was adopted as the continuous dependent variable. The mean and standard deviation (SD) were calculated for each genotype. Due to the unknown distribution of the data, Kruskal–Wallis with Dun's post‐test was used for univariate analysis. For multivariate analysis, statistical tests were adjusted to account for multiple observations per participant, given the interdependence of the data (i.e., multiple teeth from the same patient). We utilized the “Complex Samples” module in IBM SPSS for Windows version 25.0 (IBM, Chicago, IL, USA). This module accounts for elements of complex sampling designs and employs appropriate statistical techniques to handle multiple observations per participant, ensuring accurate parameter estimates and variance estimates. In brief, an analysis of variance (ANOVA) was initially conducted considering the sample design, including equal probability and PPS (probability proportional to size) methods, with or without sampling procedures. Subsequently, the results were adjusted for biological sex using a generalized linear model (GLM), with a significance level of 5%.

To minimize the risk of Type I (false‐positive) errors due to multiple comparisons, a Bonferroni adjustment was applied based on the number of independent genetic variants analyzed (0.05/6 SNPs), setting the threshold for corrected statistical significance at *p*_adjusted_ < 0.0083, in accordance with genetic association reporting standards. Associations presenting *p*‐values between 0.0083 and 0.05 were reported.

## 3 Results

From the original cohort of 143 patients (Cunha et al. 2021b) (mean age: 15.2; SD = 7.3 years; 48.3% males, 51.7% females), 35 patients were excluded based on predefined clinical criteria. The final included sample comprised 108 patients (mean age: = 15.77; SD 5.43 years; 53 males [49.1%], 55 females [50.9%]). Excluded patients (*n* = 35; 16 males, 19 females, mean age = 13.4, SD = 10.7 years) showed no significant demographic differences compared to the included group. Figure 1 illustrates the reasons and number of excluded patients in this study.

**Figure 1:**
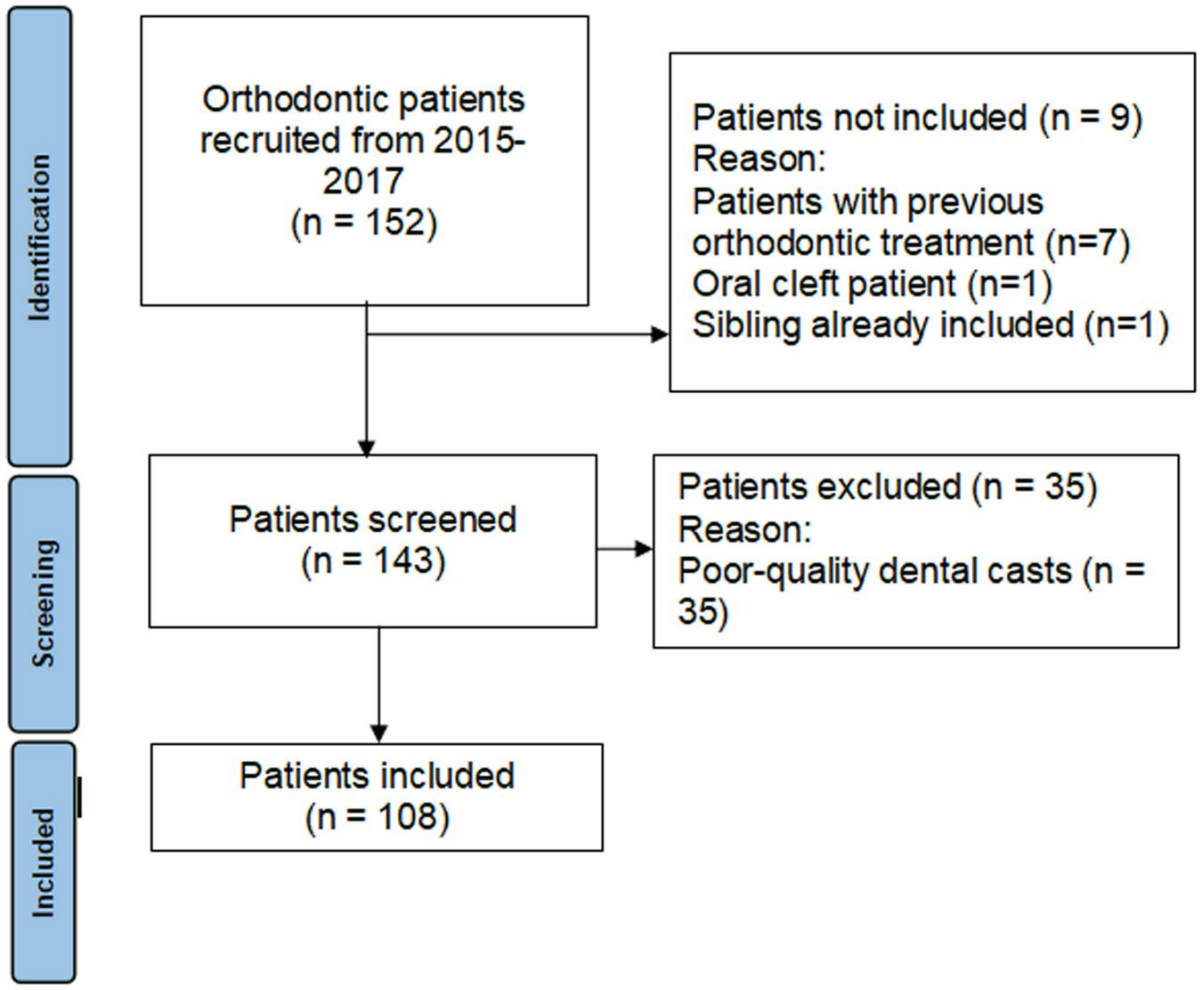
Patients screening diagram flow.

Table 1 provides details on the number of patients per genotype, genotyping success rate, Hardy–Weinberg equilibrium results, and pertinent information about the studied SNPs.

In univariate analysis (Table 2), heterozygous patients in rs694 (*PTH*) exhibited a smaller mesiodistal size of the maxillary left canine compared to dominant homozygous patients (*p* = 0.015); however, this association did not remain statistically significant after applying the Bonferroni correction (*p*_adjusted_ > 0.0083).

**Table 2:** Comparisons of means of mesiodistal measurements between genotypes for each SNP.

Tooth	Side	Maxillary teeth (SD)				Mandibular teeth (SD)			
		rs7975232 (*VDR*)							
		CC	CA	AA	*p*‐value	CC	CA	AA	*p*‐value
Central incisor	Right	8.98 (0.66)	8.73 (0.61)	8.89 (0.78)	0.139	5.55 (0.47)	5.58 (0.43)	5.62 (0.31)	0.509
	Left	9.02 (0.67)	8.82 (0.65)	8.87 (0.85)	0.433	5.51 (0.43)	5.54 (0.46)	5.56 (0.47)	0.790
Lateral incisor	Right	6.79 (0.87)	7.01 (0.79)	6.79 (0.69)	0.670	6.07 (0.47)	6.11 (0.50)	6.13 (0.33)	0.896
	Left	6.86 (0.81)	6.92 (0.60)	6.72 (0.66)	0.729	6.04 (0.46)	6.05 (0.48)	6.04 (0.44)	0.944
Canine	Right	7.68 (0.74)	7.82 (0.50)	7.76 (0.53)	0.656	6.73 (0.64)	6.87 (0.55)	7.03 (0.43)	0.147
	Left	7.64 (0.68)	7.85 (0.52)	7.81 (0.41)	0.366	6.78 (0.64)	6.96 (0.51)	6.91 (0.54)	0.321
First premolar	Right	7.09 (0.64)	7.27 (0.57)	7.15 (0.40)	0.400	7.25 (0.81)	7.23 (0.48)	7.24 (0.46)	0.920
	Left	7.07 (0.62)	7.08 (0.45)	7.37 (0.79)	0.378	7.24 (0.71)	7.31 (0.47)	7.20 (0.59)	0.854
Second premolar	Right	6.77 (0.77)	6.72 (0.78)	6.61 (0.50)	0.942	7.26 (1.10)	7.13 (1.03)	7.10 (0.48)	0.877
	Left	6.83 (0.88)	6.81 (0.81)	6.55 (0.44)	0.703	7.39 (1.11)	7.23 (0.96)	7.17 (0.64)	0.724
First Molar	Right	9.85 (0.71)	9.79 (0.58)	9.83 (0.50)	0.935	11.0 (0.73)	10.8 (0.79)	10.9 (0.74)	0.832
	Left	9.79 (0.82)	9.74 (0.59)	9.71 (0.57)	0.897	11.0 (0.67)	11.0 (0.76)	10.7 (0.68)	0.737

		rs927650 **(** ***CYP24A1*** **)**							
		**CC**	**CT**	**TT**	***p*‐value**	**CC**	**CT**	**TT**	***p*‐value**
Central incisor	Right	8.81 (0.55)	8.92 (0.69)	8.94 (0.53)	0.513	5.58 (0.41)	5.55 (0.44)	5.88 (0.39)	0.121
	Left	8.87 (0.59)	8.98 (0.71)	9.02 (0.66)	0.542	5.56 (0.42)	5.53 (0.45)	5.70 (0.51)	0.680
Lateral incisor	Right	6.80 (0.56)	6.93 (0.96)	7.29 (0.56)	0.158	6.01 (0.43)	6.16 (0.48)	6.39 (0.44)	0.170
	Left	6.84 (0.50)	6.90 (0.79)	7.12 (0.51)	0.481	5.97 (0.45)	6.11 (0.47)	6.25 (0.39)	0.318
Canine	Right	7.78 (0.51)	7.77 (0.60)	7.98 (0.62)	0.706	6.88 (0.52)	6.81 (0.54)	6.95 (0.95)	0.848
	Left	7.72 (0.54)	7.75 (0.63)	8.05 (0.41)	0.308	6.95 (0.52)	6.80 (0.56)	7.19 (0.61)	0.208
First premolar	Right	7.10 (0.62)	7.27 (0.56)	7.17 (0.57)	0.419	7.16 (0.65)	7.32 (0.64)	7.36 (0.35)	0.505
	Left	7.11 (0.62)	7.16 (0.58)	7.15 (0.39)	0.863	7.22 (0.59)	7.28 (0.62)	7.52 (0.43)	0.287
Second premolar	Right	6.56 (0.64)	6.85 (0.75)	6.85 (1.04)	0.184	7.00 (0.71)	7.32 (1.15)	7.41 (1.20)	0.196
	Left	6.54 (0.51)	6.97 (0.92)	6.94 (0.84)	0.108	7.31 (0.95)	7.36 (1.04)	7.07 (0.61)	0.843
First molar	Right	9.79 (0.56)	9.78 (0.55)	10.0 (0.89)	0.688	10.8 (0.75)	11.0 (0.68)	11.0 (1.02)	0.754
	Left	9.63 (0.66)	9.80 (0.60)	9.96 (0.88)	0.388	10.8 (0.64)	11.0 (0.62)	11.2 (1.19)	0.445

		**rs4646536 (** ***CYP27B1*** **)**							
		**AA**	**AG**	**GG**	***p*‐value**	**AA**	**AG**	**GG**	***p*‐value**
Central incisor	Right	8.83 (0.67)	8.90 (0.64)	8.63 (0.66)	0.393	5.58 (0.42)	5.59 (0.47)	5.47 (0.32)	0.846
	Left	8.81 (0.64)	9.00 (0.74)	8.92 (0.56)	0.187	5.53 (0.43)	5.55 (0.50)	5.46 (0.28)	0.578
Lateral incisor	Right	6.89 (0.72)	6.93 (0.91)	6.95 (0.54)	0.915	6.09 (0.40)	6.15 (0.53)	5.98 (0.43)	0.622
	Left	6.87 (0.65)	6.91 (0.73)	6.94 (0.49)	0.773	6.03 (0.42)	6.12 (0.52)	5.93 (0.43)	0.427
Canine	Right	7.78 (0.57)	7.71 (0.66)	7.83 (0.59)	0.962	6.82 (0.51)	6.86 (0.60)	6.88 (0.81)	0.908
	Left	7.74 (0.53)	7.78 (0.64)	7.81 (0.48)	0.897	6.85 (0.56)	6.89 (0.55)	7.03 (0.73)	0.917
First premolar	Right	7.11 (0.42)	7.30 (0.69)	6.91 (0.59)	0.276	7.26 (0.65)	7.27 (0.63)	7.03 (0.56)	0.704
	Left	7.04 (0.44)	7.20 (0.70)	7.07 (0.56)	0.554	7.21 (0.50)	7.35 (0.67)	7.20 (0.66)	0.527
Second premolar	Right	6.76 (0.13)	6.74 (0.56)	6.50 (0.51)	0.426	7.11 (1.10)	7.24 (0.90)	7.33 (0.99)	0.606
	Left	6.87 (0.98)	6.72 (0.59)	6.56 (0.64)	0.665	7.20 (0.99)	7.44 (1.06)	7.06 (0.43)	0.445
First molar	Right	9.82 (0.50)	9.84 (0.65)	9.73 (1.04)	0.677	10.9 (0.81)	10.9 (0.63)	11.1 (1.03)	0.967
	Left	9.74 (0.67)	9.75 (0.66)	9.88 (0.90)	0.974	11.0 (0.71)	10.9 (0.65)	11.1 (1.02)	0.884

		**rs694 (** ***PTH*** **)**							
		**CC**	**CT**	**TT**	***p*‐value**	**CC**	**CT**	**TT**	***p*‐value**
Central incisor	Right	8.82 (0.59)	8.85 (0.64)	9.05 (0.53)	0.331	5.47 (0.45)	5.63 (0.46)	5.63 (0.31)	0.269
	Left	8.87 (0.67)	8.88 (0.65)	9.22 (0.54)	0.117	5.47 (0.51)	5.54 (0.41)	5.68 (0.40)	0.281
Lateral incisor	Right	6.80 (0.71)	6.94 (0.87)	7.19 (0.54)	0.094	6.04 (0.45)	6.12 (0.46)	6.21 (0.48)	0.249
	Left	6.81 (0.66)	6.92 (0.70)	7.16 (0.41)	0.074	5.98 (0.41)	6.09 (0.48)	6.17 (0.43)	0.166
Canine	Right	7.90 (0.58)	7.66 (0.62)	7.84 (0.65)	0.303	6.78 (0.53)	6.78 (0.56)	7.13 (0.65)	0.072
	Left	**7.95 (0.53)** ^**a**^	**7.66 (0.62)** ^**b**^	7.92 (0.45)^ab^	**0.015**	6.88 (0.53)	6.81 (0.57)	7.18 (0.58)	0.089
First premolar	Right	7.10 (0.53)	7.20 (0.65)	7.36 (0.42)	0.185	7.19 (0.45)	7.27 (0.74)	7.41 (0.45)	0.182
	Left	7.01 (0.57)	7.15 (0.61)	7.30 (0.42)	0.124	7.23 (0.51)	7.28 (0.66)	7.44 (0.49)	0.305
Second premolar	Right	6.76 (0.68)	6.66 (0.68)	6.99 (0.92)	0.288	6.95 (0.69)	7.27 (1.11)	7.45 (1.10)	0.338
	Left	6.70 (0.64)	6.81 (0.86)	6.98 (0.85)	0.381	7.16 (0.73)	7.31 (1.01)	7.63 (1.18)	0.367
First molar	Right	9.91 (0.64)	9.79 (0.56)	9.80 (0.78)	0.735	11.0 (0.78)	10.9 (0.59)	11.0 (1.08)	0.760
	Left	9.76 (0.77)	9.79 (0.49)	9.79 (0.91)	0.985	11.1 (0.68)	10.9 (0.63)	10.9 (0.97)	0.563

		**rs6256 (** ***PTH*** **)**							
		**GG**	**GT**	**TT**	***p*‐value**	**GG**	**GT**	**TT**	***p*‐value**
Central incisor	Right	8.86 (0.60)	8.98 (0.65)	8.64 (0.00)	0.705	5.58 (0.45)	5.57 (0.40)	5.93 (0.00)	0.540
	Left	8.92 (0.67)	9.02 (0.61)	8.57 (0.00)	0.650	5.53 (0.46)	5.62 (0.38)	5.47 (0.00)	0.809
Lateral incisor	Right	6.93 (0.80)	7.00 (0.75)	6.57 (0.00)	0.656	6.10 (0.50)	6.15 (0.36)	6.32 (0.00)	0.797
	Left	6.88 (0.63)	7.07 (0.72)	6.74 (0.00)	0.717	6.05 (0.47)	6.12 (0.40)	6.02 (0.00)	0.944
Canine	Right	7.74 (0.66)	7.79 (0.45)	8.58 (0.00)	0.342	6.80 (0.64)	6.96 (0.37)	7.30 (0.00)	0.243
	Left	7.75 (0.61)	7.86 (0.41)	8.84 (0.00)	0.172	6.89 (0.60)	6.86 (0.45)	7.98 (0.00)	0.278
First premolar	Right	7.23 (0.60)	7.08 (0.51)	7.57 (0.00)	0.469	7.30 (0.66)	7.17 (0.51)	7.46 (0.00)	0.753
	Left	7.11 (0.54)	7.22 (0.69(	7.40 (0.00)	0.659	7.32 (0.60)	7.18 (0.59)	7.74 (0.00)	0.425
Second premolar	Right	6.79 (0.75)	6.71 (0.71)	6.78 (0.00)	0.892	7.30 (1.14)	6.95 (0.41)	7.39 (0.00)	0.315
	Left	6.86 (0.82)	6.68 (0.75)	7.29 (0.0)	0.206	7.42 (1.10)	7.03 (0.44)	7.66 (0.00)	0.296
First molar	Right	9.85 (0.67)	9.73 (0.51)	9.73 (0.0)	0.808	10.9 (0.83)	11.0 (0.53)	11.4 (0.00)	0.423
	Left	9.79 (0.71)	9.82 (0.43)	8.00 (0.0)	0.260	11.0 (0.79)	10.9 (0.52)	11.2 (0.00)	0.897

		**rs307247 (** ***PTH*** **)**							
		**GG**	**GA**	**AA**	***p*‐value**	**GG**	**GA**	**AA**	***p*‐value**
Central incisor	Right	8.90 (0.56)	8.83 (0.71)	8.96 (0.51)	0.871	5.57 (0.49)	5.61 (0.44)	5.60 (0.26)	0.807
	Left	8.92 (0.61)	8.89 (0.73)	9.13 (0.54)	0.426	5.54 (0.51)	5.49 (0.39)	5.67 (0.30)	0.316
Lateral incisor	Right	6.98 (0.81)	6.89 (0.81)	7.11 (0.57)	0.635	6.08 (0.47)	6.17 (0.42)	6.14 (0.54)	0.514
	Left	6.89 (0.63)	6.97 (0.71)	7.12 (0.42)	0.284	6.04 (0.47)	6.12 (0.43)	6.10 (0.47)	0.669
Canine	Right	7.87 (0.59)	7.62 (0.61)	7.82 (0.71)	0.301	6.84 (0.54)	6.80 (0.62)	7.01 (0.64)	0.435
	Left	7.84 (0.59)	7.71 (0.63)	7.90 (0.51)	0.276	6.90 (0.58)	6.87 (0.60)	7.05 (0.53)	0.591
First premolar	Right	7.22 (0.54)	7.15 (0.68)	7.31 (0.44)	0.383	7.32 (0.59)	7.27 (0.71)	7.27 (0.45)	0.843
	Left	7.07 (0.53)	7.18 (0.66)	7.27 (0.49)	0.440	7.33 (0.44)	7.26 (0.26)	7.40 (0.53)	0.590
Second premolar	Right	6.82 (0.76)	6.60 (0.54)	6.96 (1.07)	0.414	7.15 (1.13)	7.23 (0.73)	7.43 (1.29)	0.773
	Left	6.86 (0.85)	6.72 (0.67)	6.94 (0.98)	0.597	7.23 (0.99)	7.35 (0.76)	7.64 (1.40)	0.524
First molar	Right	9.87 (0.59)	9.87 (0.69)	9.60 (0.59)	0.404	11.0 (0.71)	10.9 (0.72)	10.8 (1.05)	0.818
	Left	9.80 (0.66)	9.87 (0.62)	9.52 (0.23)	0.409	11.1 (0.62)	10.9 (0.13)	10.6 (0.88)	0.174

*Note:* Kruskal–Wallis test with Dun posttest were performed. Bold forms indicate statistical difference. The letters indicate the different means.

Abbreviations: SD, standard deviation; SNP, Single Nucleotide Polymorphism.

In multivariate analysis (Table 3), the associations under the *p*_adjusted_ value (0.0083) were the following: the heterozygous genotype (CA) in rs7975232 (*VDR*) was associated with a smaller mesiodistal size of the right (*β* = −0.39; *p* = 0.001) and left (*β* = −0.37; *p* = 0.004) maxillary central incisors in comparison to the dominant homozygous genotype (CC). The recessive homozygous genotype in rs6256 (*PTH*) was associated with smaller mesiodistal size of the left maxillary first molar (*β* = −2.01; *p* = 0.001).

**Table 3:** General linear model of measurements between genotypes of each SNPs.

Genetic markers				Central incisor				Lateral incisor				Canine				First premolar				Second premolar				First molar			
				Right		Left		Right		Left		Right		Left		Right		Left		Right		Left		Right		Left	
Gene	SNP	Reference	Genotype	*β* [95% CI]	*p*‐value	*β* [95% CI]	*p*‐value	*β* [95% CI]	*p*‐value	*β* [95% CI]	*p*‐value	*β* [95% CI]	*p*‐value	*β* [95% CI]	*p*‐value	*β* [95% CI]	*p*‐value	*β* [95% CI]	*p*‐value	β [95% CI]	*p*‐value	*β* [95% CI]	*p*‐value	*β* [95% CI]	*p*‐value	*β* [95% CI]	*p*‐value
**Maxillary teeth**																											
VDR	rs7975232	CC	CA	**−0.39 [−0.62, −0.16]**	**0.001**	**−0.37 [−0.62, −0.12]**	**0.004**	0.00 [−0.24, 0.24]	0.974	−0.06 [−0.30, 0.18]	0.621	0.03 [−0.21, 0.27]	0.778	0.09 [−0.15, 0.33]	0.463	0.10 [−0.14, 0.34]	0.417	−0.17 [−0.41, 0.07]	0.180	−0.19 [−0.43, 0.05]	0.211	−0.06 [−0.30, 0.18]	0.714	−0.12 [−0.36, 0.12]	0.290	−0.11 [−0.35, 0.13]	0.419
			AA	0.26 [−0.17, 0.69]	0.229	0.04 [−0.39, 0.47]	0.863	0.05 [−0.38, 0.48]	0.851	−0.02 [−0.45, 0.41]	0.918	−0.08 [−0.51, 0.35]	0.689	0.11 [−0.32, 0.54]	0.607	0.09 [−0.34, 0.52]	0.682	**0.61 [0.18– 1.04]**	**0.040**	−0.04 [−0.47, 0.39]	0.853	−0.11 [−0.54, 0.32]	0.702	0.11 [−0.32, 0.54]	0.592	0.27 [−0.16, 0.70]	0.247
CYP24A1	rs927650	CC	CT	0.21 [−0.12, 0.54]	0.873	0.03 [−0.30, 0.36]	0.773	0.04 [−0.29, 0.37]	0.776	—	—	−0.05 [−0.38, 0.28]	0.679	−0.01 [−0.34, 0.32]	0.940	0.13 [−0.20, 0.46]	0.300	0.01 [−0.32, 0.34]	0.928	0.20 [−0.13, 0.53]	0.182	0.30 [−0.03, 0.63]	0.077	−0.06 [−0.39, 0.27]	0.611	0.17 [−0.16, 0.50]	0.204
			TT	0.05 [−0.44, 0.54]	0.812	0.07 [−0.42, 0.56]	0.737	0.33 [−0.16, 0.82]	0.216	0.17 [−0.32, 0.66]	0.447	0.20 [−0.29, 0.69]	0.320	0.27 [−0.22, 0.76]	0.188	−0.04 [−0.53, 0.45]	0.837	−0.01 [−0.50, 0.48]	0.947	0.06 [−0.43, 0.55]	0.806	0.09 [−0.40, 0.58]	0.753	0.26 [−0.23, 0.75]	0.193	0.22 [−0.27, 0.71]	0.333
CYP27B1	rs4646536	AA	AG	0.08 [−0.16, 0.32]	0.489	0.18 [−0.06, 0.42]	0.162	−0.03 [−0.27, 0.21]	0.808	−0.00 [−0.24, 0.24]	0.990	−0.06 [−0.30, 0.18]	0.603	−0.00 [−0.24, 0.24]	0.960	**0.25 [0.01, 0.49]**	**0.040**	0.19 [−0.05, 0.43]	0.132	0.06 [−0.18, 0.30]	0.691	−0.09 [−0.33, 0.15]	0.592	0.04 [−0.20, 0.28]	0.733	−0.07 [−0.31, 0.17]	0.616
			GG	−0.36 [−0.78, 0.06]	0.094	−0.04 [−0.46, 0.38]	0.843	−0.19 [−0.61, 0.23]	0.945	−0.11 [−0.53, 0.31]	0.616	−0.01 [−0.43, 0.41]	0.960	0.00 [−0.42, 0.42]	0.999	−0.40 [−0.81, 0.01]	0.056	−0.10 [−0.52, 0.32]	0.619	−0.40 [−0.82, 0.02]	0.124	−0.44 [−0.86, −0.02]	0.133	−0.32 [−0.74, 0.10]	0.115	−0.06 [−0.48, 0.36]	0.798
PTH	rs694	CC	CT	0.04 [−0.20, 0.28]	0.715	0.05 [−0.19, 0.29]	0.971	0.11 [−0.13, 0.35]	0.482	0.10 [−0.14, 0.34]	0.422	−0.04 [−0.28, 0.20]	0.722	−0.21 [−0.45, 0.03]	0.092	0.08 [−0.16, 0.32]	0.500	0.09 [−0.15, 0.33]	0.469	−0.08 [−0.32, 0.16]	0.564	0.08 [−0.16, 0.32]	0.658	0.04 [−0.20, 0.28]	0.744	0.15 [−0.09, 0.39]	0.276
			TT	0.05 [−0.25, 0.35]	0.719	0.14 [−0.16, 0.44]	0.386	0.09 [−0.21, 0.39]	0.640	0.07 [−0.23, 0.37]	0.673	−0.09 [−0.39, 0.21]	0.533	0.04 [−0.26, 0.34]	0.794	0.09 [−0.21, 0.39]	0.565	0.12 [−0.18, 0.42]	0.447	0.06 [−0.24, 0.36]	0.727	0.02 [−0.28, 0.32]	0.929	**−0.30 [−0.60, −0.00]**	**0.047**	−0.22 [−0.52, 0.08]	0.209
	rs6256	GG	GT	0.10 [−0.22, 0.42]	0.477	0.08 [−0.24, 0.40]	0.571	0.12 [−0.20, 0.44]	0.500	0.25 [−0.07, 0.57]	0.778	−0.00 [−0.32, 0.32]	0.967	0.07 [−0.25, 0.39]	0.607	−0.21 [−0.53, 0.11]	0.158	0.06 [−0.26, 0.38]	0.665	−0.10 [−0.42, 0.22]	0.556	−0.17 [−0.49, 0.15]	0.417	−0.08 [−0.40, 0.24]	0.584	0.07 [−0.25, 0.39]	0.639
			TT	−0.46 [−1.66, 0.74]	0.434	−0.67 [−1.87, 0.53]	0.268	−0.71 [−1.91, 0.49]	0.336	−0.42 [−1.62, 0.78]	0.493	0.61 [−0.59, 1.81]	0.266	0.92 [−0.28, 2.12]	0.107	0.21 [−0.99, 1.41]	0.713	0.12 [−1.08, 1.32]	0.829	−0.28 [−1.48, 0.92]	0.693	0.30 [−0.90, 1.50]	0.705	−0.32 [−1.52, 0.88]	0.562	**−2.01 [−3.21, −0.81]**	**0.001**
	rs307247	GG	GA	−0.03 [−0.35, 0.29]	0.817	−0.00 [−0.32, 0.32]	0.953	−0.03 [−0.35, 0.29]	0.846	0.08 [−0.24, 0.40]	0.514	−0.16 [−0.48, 0.16]	0.186	−0.08 [−0.40, 0.24]	0.505	−0.04 [−0.36, 0.28]	0.717	0.09 [−0.23, 0.41]	0.492	−0.24 [−0.56, 0.08]	0.121	−0.19 [−0.51, 0.13]	0.284	0.07 [−0.25, 0.39]	0.534	0.17 [−0.15, 0.49]	0.220
			AA	0.00 [−0.35, 0.35]	0.959	0.10 [−0.25, 0.45]	0.580	0.06 [−0.29, 0.41]	0.768	0.10 [−0.25, 0.45]	0.576	−0.02 [−0.37, 0.33]	0.885	0.06 [−0.29, 0.41]	0.710	0.08 [−0.27, 0.43]	0.646	0.10 [−0.25, 0.45]	0.567	0.11 [−0.24, 0.46]	0.580	0.04 [−0.31, 0.39]	0.844	−0.31 [−0.63, 0.01]	0.058	−0.34 [−0.71, 0.03]	0.073
**Mandibular teeth**																											
VDR	rs7975232	CC	CA	−0.08 [−0.36, 0.20]	0.394	−0.07 [−0.35, 0.21]	0.417	−0.06 [−0.34, 0.22]	0.547	−0.09 [−0.37, 0.19]	0.353	−0.09 [−0.37, 0.19]	0.432	−0.01 [−0.29, 0.27]	0.878	−0.14 [−0.42, 0.14]	0.284	−0.05 [−0.33, 0.23]	0.698	−0.26 [−0.54, 0.02]	0.252	−0.31 [−0.59, −0.03]	0.143	**−0.33 [−0.61, −0.05]**	**0.021**	−0.10 [−0.38, 0.18]	0.494
			AA	0.16 [−0.25, 0.57]	0.310	0.10 [−0.31, 0.51]	0.515	0.05 [−0.36, 0.46]	0.737	0.17 [−0.24, 0.58]	0.291	0.29 [−0.12, 0.70]	0.121	0.10 [−0.31, 0.51]	0.576	0.13 [−0.28, 0.54]	0.562	0.14 [−0.27, 0.55]	0.508	0.01 [−0.40, 0.42]	0.970	−0.08 [−0.49, 0.33]	0.804	0.02 [−0.39, 0.43]	0.903	−0.28 [−0.69, 0.13]	0.264
CYP24A1	rs927650	CC	CT	−0.09 [−0.29, 0.11]	0.318	−0.05 [−0.25, 0.15]	0.569	0.05 [−0.15, 0.25]	0.552	0.07 [−0.13, 0.27]	0.447	−0.06 [−0.26, 0.14]	0.573	−0.17 [−0.37, 0.03]	0.095	0.05 [−0.15, 0.25]	0.676	−0.02 [−0.22, 0.18]	0.820	0.21 [0.01, 0.41]	0.343	0.11 [−0.09, 0.31]	0.593	0.09 [−0.11, 0.29]	0.525	0.03 [−0.17, 0.23]	0.819
			TT	0.31 [−0.01, 0.63]	0.056	0.15 [−0.19, 0.49]	0.346	0.29 [−0.04, 0.62]	0.086	0.19 [−0.15, 0.53]	0.269	0.10 [−0.24, 0.44]	0.585	0.30 [−0.05, 0.65]	0.091	0.07 [−0.27, 0.41]	0.754	0.21 [−0.13, 0.55]	0.305	0.14 [−0.20, 0.48]	0.722	−0.37 [−0.71, −0.03]	0.318	0.04 [−0.30, 0.38]	0.840	0.42 [−0.06, 0.90]	0.087
CYP27B1	rs4646536	AA	AG	0.01 [−0.26, 0.28]	0.892	0.03 [−0.24, 0.30]	0.702	0.10 [−0.17, 0.37]	0.276	0.09 [−0.18, 0.36]	0.335	0.05 [−0.22, 0.32]	0.632	−0.00 [−0.27, 0.27]	0.978	0.03 [−0.24, 0.30]	0.817	0.10 [−0.17, 0.37]	0.425	0.10 [−0.17, 0.37]	0.638	0.26 [−0.01, 0.53]	0.216	0.02 [−0.25, 0.29]	0.858	−0.04 [−0.31, 0.23]	0.759
			GG	−0.08 [−0.54, 0.38]	0.612	−0.05 [−0.51, 0.41]	0.754	−0.19 [−0.65, 0.27]	0.247	−0.20 [−0.66, 0.26]	0.215	−0.05 [−0.51, 0.41]	0.765	0.06 [−0.40, 0.52]	0.746	−0.39 [−0.85, 0.07]	0.098	−0.19 [−0.65, 0.27]	0.360	0.09 [−0.37, 0.55]	0.807	−0.36 [−0.82, 0.10]	0.305	−0.03 [−0.49, 0.43]	0.882	−0.05 [−0.51, 0.41]	0.819
PTH	rs694	CC	CT	0.13 [−0.17, 0.43]	0.144	0.03 [−0.27, 0.33]	0.719	0.13 [−0.17, 0.43]	0.185	0.08 [−0.22, 0.38]	0.402	0.00 [−0.30, 0.30]	0.951	−0.04 [−0.34, 0.26]	0.704	0.08 [−0.22, 0.38]	0.547	0.02 [−0.28, 0.32]	0.834	0.19 [−0.11, 0.49]	0.415	0.07 [−0.23, 0.37]	0.746	0.08 [−0.22, 0.38]	0.575	0.01 [−0.29, 0.31]	0.907
			TT	−0.00 [−0.34, 0.34]	0.961	0.13 [−0.21, 0.47]	0.249	−0.01 [−0.35, 0.33]	0.906	0.01 [−0.33, 0.35]	0.893	0.15 [−0.19, 0.49]	0.283	0.11 [−0.23, 0.45]	0.396	0.01 [−0.33, 0.35]	0.946	0.01 [−0.33, 0.35]	0.908	0.17 [−0.17, 0.51]	0.538	0.19 [−0.15, 0.53]	0.469	−0.23 [−0.57, 0.11]	0.190	−0.34 [−0.68, 0.00]	0.052
	rs6256	GG	GT	−0.02 [−0.34, 0.30]	0.851	0.07 [−0.25, 0.39]	0.531	0.03 [−0.29, 0.35]	0.740	0.10 [−0.22, 0.42]	0.365	0.13 [−0.19, 0.45]	0.311	−0.05 [−0.37, 0.27]	0.668	−0.13 [−0.45, 0.19]	0.410	−0.09 [−0.41, 0.23]	0.518	−0.34 [−0.66, −0.02]	0.205	−0.40 [−0.72, −0.08]	0.103	0.09 [−0.23, 0.41]	0.573	−0.05 [−0.37, 0.27]	0.742
			TT	0.20 [−1.08, 1.48]	0.642	−0.20 [−1.48, 1.08]	0.648	0.00 [−1.28, 1.28]	0.984	−0.20 [−1.48, 1.08]	0.648	0.18 [−1.10, 1.46]	0.731	0.79 [−0.49, 2.07]	0.107	0.01 [−1.27, 1.29]	0.987	0.23 [−1.05, 1.51]	0.687	−0.00 [−1.28, 1.28]	0.994	0.01 [−1.27, 1.29]	0.984	0.14 [−1.14, 1.42]	0.829	−0.04 [−1.32, 1.24]	0.950
	rs307247	GG	GA	0.08 [−0.24, 0.40]	0.409	−0.05 [−0.37, 0.27]	0.593	0.14 [−0.18, 0.46]	0.164	0.11 [−0.21, 0.43]	0.251	0.01 [−0.31, 0.33]	0.893	0.04 [−0.28, 0.36]	0.722	−0.01 [−0.33, 0.31]	0.946	−0.06 [−0.38, 0.26]	0.624	0.03 [−0.29, 0.35]	0.877	0.05 [−0.27, 0.37]	0.800	0.10 [−0.22, 0.42]	0.511	0.02 [−0.30, 0.34]	0.892
			AA	−0.05 [−0.41, 0.31]	0.686	0.09 [−0.27, 0.45]	0.475	−0.05 [−0.41, 0.31]	0.665	−0.03 [−0.39, 0.33]	0.795	0.09 [−0.27, 0.45]	0.541	0.06 [−0.30, 0.42]	0.679	−0.08 [−0.44, 0.28]	0.635	0.03 [−0.33, 0.39]	0.839	0.17 [−0.19, 0.53]	0.587	0.24 [−0.12, 0.60]	0.399	−0.28 [−0.64, 0.08]	0.140	**−0.47 [−0.83, −0.11]**	**0.011**

Abbreviations: CI, confidence interval; SNP, Single Nucleotide Polymorphism.

The associations with *p*‐values between 0.0083 and 0.05 in multivariate analysis (Table 3) were as follows: the heterozygous genotype (CA) at rs7975232 (VDR) was associated with a smaller mesiodistal size of the right mandibular first molar (*β* = −0.33; *p* = 0.021). The recessive homozygous genotype of the same SNP (AA) was associated with a greater mesiodistal size of the maxillary left first premolar compared to the dominant homozygous genotype (*β* = 0.61; *p* = 0.040). The heterozygous genotype (AG) at rs4646536 (CYP27B1) was associated with a greater mesiodistal size of the maxillary right first premolar (*β* = 0.25; *p* = 0.040) than the dominant homozygous genotype (AA). The recessive homozygous genotype in rs694 (*PTH*) was associated with smaller mesiodistal size of the right maxillary first molar (*β* = −0.30; *p* = 0.047). The recessive homozygous genotype (GG) in rs307247 (*PTH*) was also associated with smaller mesiodistal size of the right maxillary first molar (*β* = −0.47; *p* = 0.011).

## 4 Discussion

The results of our study suggest the significant impact of SNPs in MTS and support the hypothesis that genes involved indirectly in odontogenesis may be potential risk factors for dental malocclusions (Jabri MA et al. 2019; Doris et al. 1981; Murugesan and Sivakumar 2020). Here, we evaluated genes involved in calcium and vitamin D homeostasis. Calcium, vital for tooth formation, and vitamin D, crucial for calcium absorption, are essential for healthy tooth development. The calcium‐sensing receptor in human odontogenic cells induces odontoblastic differentiation in response to elevated calcium concentrations (Mizumachi et al. 2017). Inadequate vitamin D availability leads to hypocalcemia and impaired tooth development. Vitamin D also regulates the expression of genes involved in dentin and enamel mineralization, thereby fostering proper tooth formation 9. Hence, it stands to reason that disruptions in the availability of calcium and vitamin D may impair tooth development. These disruptions can arise from various factors, including diet (Lips et al. 2014), diseases (Al‐Ghafari et al. 2019; Ahn et al. 2009), and SNPs (Krasniqi et al. 2021). In our study, we rejected the null hypothesis due to the association between MTS and SNPs in *VDR* (rs7975232) and *PTH* (rs6256) genes. While these genetic variations are associated with tooth size in our cohort, caution is warranted. At this stage, these SNPs cannot be established as direct clinical biomarkers to predict extraction needs or treatment pathways. Instead, they should be viewed as preliminary candidate risk factors that help elucidate the polygenic etiology of tooth‐size discrepancies and require further validation in larger, independent prospective studies before clinical translation can be proposed.

We opted to measure MTS over other dental dimensions for several reasons: the mesiodistal dimension is less affected by natural tooth wear due to aging, as well as non‐natural factors such as bruxism, malocclusions, and harmful habits; besides that, the mesiodistal dimension plays a crucial role in various dental procedures, particularly in orthodontics. In orthodontic treatment planning, determining the MTS of permanent teeth and the available space for tooth alignment is of critical importance (Doris et al. 1981). We used dental casts to measure the MTS, which may be a limitation of our study. Digital models from 3D scanning or cone‐beam tomography provide more accurate measurements with a lower risk of distortion. However, dental casts remain the most commonly used method worldwide for orthodontic treatment planning (Murugesan and Sivakumar 2020).

VDR is expressed in odontoblasts, ameloblasts, and cementoblasts in human tooth germs (Bailleul‐Forestier et al. 1996), contributing to dentin and enamel mineralization (Landin et al. 2012; Zhang et al. 2009, 2007). We observed that the heterozygous (CA) genotype at rs7975232 (also known as ApaI) in the VDR gene affects the mesiodistal size of the maxillary central incisors and mandibular first molars. Interestingly, the heterozygous genotype for ApaI was linked with lower plasma levels of calcium (Al‐Ghafari et al. 2019). It is postulated that ApaI plays a role in stabilizing VDR‐Mrna (Durrin et al. 1999), enhancing gene transcription, and modifying cellular responses to circulating calcium and vitamin D (Uitterlinden et al. 2004). Thus, we posit that ApaI may regulate tooth size by altering the response of odontogenesis‐related cells to circulating calcium and vitamin D.

We also investigated SNPs in genes encoding two crucial enzymes in vitamin D metabolism: CYP27B1 and CYP24A1. CYP27B1 orchestrates the conversion of the primary circulating form of vitamin D (25‐hydroxyvitamin D (25(OH)D)) into its biologically active form (1,25‐dihydroxyvitamin D (1,25(OH)2D)), which then binds to VDR to regulate the expression of numerous genes within cellular nuclei. Conversely, CYP24A1 prevents the buildup of excess circulating vitamin D, which can lead to hypercalcemia, hyperphosphatemia, and toxicity (Prosser and Jones 2004). We observed that the heterozygous genotype (AG) at the rs4646536 (CYP27B1) SNP was associated with greater mesiodistal size of the maxillary first premolar. Since CYP27B1 is expressed in human odontoblasts (van der Meijden et al. 2016), and rs4646536 is an intronic variant, it may also be linked to gene transcription levels. The heterozygous genotype of the rs4646536 SNP was associated with increased vitamin D levels (Yu et al. 2019). Our team's previous study also demonstrated that this genotype protects against mandibular retrognathism (Küchler et al. 2021). Thus, it is reasonable to imply that the AG genotype of rs4646536 SNP in the *CYP27B1* gene may heighten circulating vitamin D during odontogenesis, consequently augmenting tooth size, as well as enhancing the growth potential of the mandible, according to our study (Küchler et al. 2021). However, as this association did not reach the threshold for Bonferroni correction (*p*_adjusted_ = 0.083), these results must be interpreted with caution as preliminary and warrant validation in independent cohorts.

PTH facilitates the reabsorption of calcium from bone cells to elevate circulating calcium levels (Khundmiri et al. 2016). Additionally, odontoblasts express PTH receptor 1, which modulates proliferative and apoptotic cellular responses (Sun et al. 2010; Guimarães et al. 2014). Alterations in serum PTH levels during odontogenesis can affect dentine synthesis and mineralization. In our study, we studied some SNPs in the *PTH* gene (rs694, rs307247, and rs6256) previously associated with low serum PTH levels (Ertl et al. 2012; Kanzawa et al. 1999; Tenne et al. 2008; Hibler et al. 2015). We hypothesize that recessive genotypes at these SNPs reduce PTH synthesis, thereby compromising the resorption and availability of circulating calcium during odontogenesis and modifying the MTS of permanent teeth. Our findings regarding the *PTH* gene on MTS should be interpreted with caution: the rs694 and rs307247 SNPs did not reach significance after Bonferroni correction and were not in Hardy–Weinberg equilibrium. Although the Ensembl global database reports an MAF > 0.30 for these variants, the HWE deviation in our sample likely reflects population stratification, characteristic of highly admixed populations and attributable to the convenience sample. Additionally, while the association of rs6256 (PTH) with a smaller left maxillary first molar achieved statistical significance within our model after Bonferroni correction, only one patient in our sample had this homozygous recessive genotype (*N* = 1). Consequently, this finding is highly susceptible to individual anatomical variation or developmental anomalies unique to this single subject. Therefore, it is premature to conclude a definitive biological trend from a single individual. This result should be treated strictly as an isolated descriptive observation.

## 5 Conclusion

In conclusion, our exploratory findings suggest that genetic polymorphisms in *VDR* and PTH are associated with variations in the dimensions of permanent tooth crowns. While these results contribute to our understanding of the genetic basis of odontogenesis, they must be interpreted with caution. Given the exploratory and cross‐sectional nature of this study, future multicenter, prospective investigations in larger, ethnically diverse cohorts are necessary to validate these associations before clinical screening applications can be considered.

## Author Contributions

Conceptualization: Mirian Aiko Nakane Matsumoto, Maria Bernadete Sasso Stuani, Christian Kirschneck, and Erika Calvano Küchler. Methodology: Guido Artemio Marañón‐Vásquez, Fábio Lourenço Romano, and Erika Calvano Küchler. Software: Caio Luiz Bitencourt Reis. Validation: Daniela Silva Barroso de Oliveira, Juliane Cora da Silva, and Flares Baratto‐Filho. Formal analysis: Caio Luiz Bitencourt Reis and Erika Calvano Küchler. Investigation: Caio Luiz Bitencourt Reis. Resources: Mirian Aiko Nakane Matsumoto, Maria Bernadete Sasso Stuani, Christian Kirschneck and Erika Calvano Küchler. Data curation: Caio Luiz Bitencourt Reis. Writing – original draft preparation: Caio Luiz Bitencourt Reis, and Erika Calvano Küchler. Writing – review and editing: Caio Luiz Bitencourt Reis, Mirian Aiko Nakane Matsumoto, Maria Bernadete Sasso Stuani, Fábio Lourenço Romano, Guido Artemio Marañón‐Vásquez, Daniela Silva Barroso de Oliveira, Flares Baratto‐Filho, Juliane Cora da Silva, Christian Kirschneck, and Erika Calvano Küchler. Visualization: Erika Calvano Küchler. Supervision: Mirian Aiko Nakane Matsumoto. Project administration: Mirian Aiko Nakane Matsumoto, Maria Bernadete Sasso Stuani, Christian Kirschneck, Erika Calvano Küchler. Funding acquisition: Christian Kirschneck and Erika Calvano Küchler.

## Ethics Statement

The Human Ethics Committee of the School of Dentistry of Ribeirão Preto, University of São Paulo, Brazil (Register: 01451418.3.0000.5419/3.150.551) approved this study.

## Consent

All patients included were previously informed of the written consent.

## Conflicts of Interest

The authors declare no conflicts of interest.

## Declaration

The authors declare that they did not use AI and AI‐assisted technologies in the writing process.

## Supporting information


Supporting File

## Data Availability

The data sets supporting the conclusions of this article are included within the article. We provided in the supplementary material section the exact base pair to each patient per polymorphism in an.xls archive file.
